# The imaginative failure of normal: Considerations for a post-pandemic future

**DOI:** 10.1177/1473325020973338

**Published:** 2021-03

**Authors:** Sandra M Leotti

**Affiliations:** University of Wyoming, USA

**Keywords:** Crisis, social justice, critical social work, legitimation crisis

## Abstract

The COVID-19 pandemic has struck a forceful blow to an already faltering economic and political system. Decades of neoliberal capitalism have left us with extreme domestic and global inequality, a climate disaster, and the death of democracy. Now, in the midst of a global pandemic, any solace found in the toxic status quo has all but vanished. We are faced with an event of major historical significance, the effects of which are sure to be vast and enduring. As we emerge from this crisis and rebuild, we have a tremendous opportunity to (re)consider and (re)formulate the kind of world we want to live in. This essay reflects on the limitations of an uncritical desire for a return to “normalcy.” Further, it asks social workers to engage in a process of imaginative and speculative thinking to envision possibilities for our inevitably changed world.

I write this essay from the vantage point of being a fully employed (for now), child-free, tenure track professor in a small rural town in the western United States. Unlike so many others, I am not trapped in a small apartment; I can easily access recreational opportunities and outside spaces without putting myself or others in danger; I am not immediately worried about how to pay the rent; I have yet to fall ill; and I do not have the added burden of juggling my own work while also homeschooling children or caring for the sick or elderly. While I certainly miss physical interactions that come with ease rather than anxiety, and while, like others, I shoulder the disappointments of a life on pause, I have made my peace with social distancing. Undoubtedly, the views expressed in this essay are shaped, in part, by a certain level of wellness and stability that I have managed to retain during the pandemic. However, what has become increasingly clear as time unfolds is that wellness, or stability, for any of us is precarious at best, and could easily be otherwise.

There is very real pain and loss embedded in this historical moment. People are dying, and they are dying alone. Deep economic insecurity has captured so many with no real end in sight. The fear and uncertainty can be crippling. This is a crisis to be sure, but what if it’s more than that? What if it is also an opportunity? Global catastrophes create the conditions for breaking with the past and building the world anew. The current pandemic is no exception. Roy (2020) describes the pandemic as a “portal”: an opening, a way into a new era. As many of us have been forced to slow down and find new ways to move through the world, I suggest that rather than rushing to resume our lives as if this crisis never happened, we take the time to examine the world that was compared to the one that now is, and use this as an opportunity to imagine a world that could be.

## Normal is not enough

Here in the United States, there is ubiquitous and continuous chatter in the media, from politicians, and among friends and colleagues about returning to normality. Throughout much of the spring, as lockdowns ensued in many parts of the country, right wing conservative citizens actively protested shelter in place orders, demanding that we quickly and urgently open the economy to “normal” activity. Brené Brown (2020), arguably one of the most public figures in US social work today, insisted that what we are experiencing is collective grief over the loss of “normal.” Proponents of the democratic presidential candidate, Joe Biden, feeding on the fear and trauma caused by the Trump administration, wrapped their platform in the need for a return to normalcy, to a past when our leaders served us and the country was less chaotic. Everyday people yearn to leave their homes to socialize, travel, and get haircuts: to get back to “normal.” The examples are endless. Certainly, the longing for economic security, daily comforts, and a national leader without fascist tendencies are reasonable desires. Yet if we look deeper, we see that the idea of normalcy that is so sought after also entails unfettered capitalism, rampant consumption, widespread health disparities, extreme economic inequality, and a disregard for planetary limits. Perhaps the unprecedented times we find ourselves in are not abnormal at all but rather an extension, and undeniable byproduct, of the coveted normal. In this sense I cannot help but wonder, who does normal serve? And, more importantly, shouldn’t we want more than that?

The call to “get back to normal” seems too quick and too easy. It troubles me, and the potential to fall back into old destructive patterns strikes fear in my heart. The collective grasp for normalcy undermines the fact that normal was not working for a large swath of our population prior to the pandemic; and it wasn’t working long before President Donald Trump took office. The hard truth is that the political and economic ground from which “normal” emerged is toxic to the environment and breeds deep domestic and global inequities. Still, there is an assumed salvific version of it that proliferates our news feeds. By way of example, let’s explore the talk about the economy.

As the US economy shuttered to a halt, the media, economists, and politicians extolled the virtues of our pre-pandemic economy and bemoaned the loss of years of job growth. The political message on the right insinuates that, pre-COVID, President Donald Trump was responsible for, and was governing over, a thriving economy. The same people suggest that our current predicament (i.e. the most extreme economic downturn of our lifetimes) is simply because of the pandemic – which no one, not even Trump himself, could control. In contrast, the dominant political message on the left suggests that the now shambles of our once prosperous economy are the sole result of the Trump administration’s failures. Regardless of the rationale, the image of a lost booming economy is tossed upon us, recklessly, in hopes that we will grab at any opportunity to pick back up where we left off and return to normal. The unpleasant reality is that such a picturesque “booming economy” is, and was, a lie. It's a lie propagated despite all the very real cracks that have been present and widening in the economy for decades: rampant under-employment, stagnant wages, declining worker power and protections, vast inequalities in wealth and health, and the overall evaporation of a middle class (Formisano, 2015). The list could go on. Then in March, we found our realities upended by a global pandemic. Things that were already bad have only gotten worse. The pandemic did not create our economic woes out of thin air; it only exposed them to the masses and then smashed down the already crumpling house of cards.

Indeed, amidst the pandemic, the failings of neoliberal capitalism and US democracy have become all too clear. Our consumer-based economy that thrives on low wage, precarious work only booms for a small fragment of our society before busting for us all, and the absence of a strong and reliable social safety net intensifies the economic and health impacts of the virus. Millions in the US are now left unemployed and disconnected from employer-based health insurance, rendered unable to meet their most basic needs for health care, food, and shelter. Communities of color are being disproportionately ravaged by COVID-19: a reflection of racial health disparities that have plagued the US for decades. And states now find themselves tumbling into unprecedented fiscal crises with federal pressure to file for bankruptcy in lieu of receiving governmental support. Nothing that has evolved over the last forty years of rampant neo-liberal globalization has prepared us to survive this moment; we have been told that the market will fix all of our woes, but the coronavirus cares not for profit, only for proliferation. The strategies of the market, which prioritize economic efficiency and gain over social and moral obligations (Brown, 2015; Wacquant, 2014), will not fix the coronavirus and they will not fix the overlapping crises we now find ourselves in. Indeed, the unmitigated allegiance to the market has only exacerbated the fallout of the pandemic around the globe. This is the exact moment when the cavalier evisceration of the public sphere fails us all.

## Staying with the trouble

It seems that we have reached what Habermas (1975) calls a “legitimation crisis.” Nancy Fraser (2015) builds on Habermas’s work and explains that when political institutions face the inevitable failures of capitalism, and thus miscarry on their promises (i.e. lies) to deliver health and prosperity, popular power (i.e. people) will turn against the system and seek its transformation in favor of the public good. I believe we have reached the legitimation crisis of our time. The COVID-19 pandemic has struck an enormous blow to an already faltering economic system, shocking many people awake from their complacency. The coveted status quo has vanished from beneath us and our interconnections and vulnerabilities are becoming visible like never before. The need for policies and government interventions that support the many, rather than the few, have become all too apparent. If meaningful change is ever going to happen, it is now. Indeed, in the context of the irreversible effects of climate change (remember, the virus is not the only crisis we face), this may be humanity’s only real hope to change course before it’s too late.

So no, I do not grieve the loss of “normal.” And, no, I do not wish to go back to the toxic normal we have existed in for as long as I have been alive on this planet. I do not long to continue course in a global economy that promotes ecological destruction and exploits the lives of so many. I want something better for myself, for our communities, and for our future. We can continue down the path we have been heading, embracing the lies of the 20^th^ century, exploiting the lives of black and brown bodies around the globe, and accelerating climate change – thus robbing future generations of any chance for health and vitality – or we can choose another way forward to a future built not on market-based values but on values of sustainability, collective well-being, and equity. A new reality is coming whether we are ready for it or not. Envisioning and creating that reality is one of social work’s most pressing obligations.

Donna Haraway (2016) implores us to “stay with the trouble.” Staying with the trouble, she urges, is not a passive acceptance of death and destruction, but rather an active engagement with the uncertain messiness of our times. Urgent times require us to think through the disorder, to not jump to easy solutions rooted in a return to an imagined idyllic past or an illusory escape to some redemptive future. Rather, she says that our task is to, “make trouble, to stir up potent response to devastating events, as well as to settle troubled waters and rebuild quiet places.” And that “staying with the trouble requires learning to be truly present … as moral critters entwined in myriad unfinished configurations of places, times, and meanings” (Haraway, 2016: 1).

Haraway offers an encouraging reminder that everyday people are not powerless. Just as the examples of overlapping system failures have flourished over the last weeks and months, so too have examples of resistance and possibility. As shelter in place orders were rolled out, low wage workers, suddenly deemed essential, felt emboldened to strike for safer working conditions and better pay. The United States Congress reached bipartisan spending agreements, extending unemployment benefits and administering direct payments to individuals. Federal and state authorities instituted widespread moratoriums on evictions and called for the instant release of non-violent prisoners. And back in March, the United Nations Secretary-General António Guterres called for a humanitarian global ceasefire in order to combat the coronavirus. In July, the UN Security Council unanimously adopted the resolution demanding immediate cessation of conflicts. What these examples teach us is that many of the social, political, and economic conditions we have come to accept as inevitable are instead the product of pointed political decision making. Ideas and actions that seemed unthinkable just a short while ago have now become a reality. Such actions may be temporary, but if this pandemic is going to be more than another crisis for capitalism to exploit, we must fight like hell against the allure of normality and revise our expectations of what we think is possible.

Clinging to the past pacifies through nostalgia and comfort, and claiming the future is no easy task. To be sure, it will be wrought with conflict and confusion, but it also comes with the possibility of moving beyond frightening certainties: ecological collapse, extreme economic and racial injustice, a hollowed-out democracy (Brown, 2015; Klein, 2017). As a profession, we have a responsibility to both reject the easy grab for “normal” and take the lead in boldly imagining a new future built on sustainable and equitable policies and practices. *And to then fight for it.* When this crisis passes, we are going to enter a vastly different world. We cannot bring back the lives that are lost, but we can try to ensure that those lives are not lost in vain. In our classrooms, in our research, and in our public conversations, let us use this as an opportunity to boldly articulate a collective vision of what could be, and to assert a renewed and urgent sense of civic and political responsibility. Let this be the animating force for our profession in the years and decades to come.
